# The Prevalence and Significance of HTLV-I/II Seroindeterminate Western Blot Patterns

**DOI:** 10.3390/v3081320

**Published:** 2011-08-02

**Authors:** Anna Abrams, Yoshimi Akahata, Steven Jacobson

**Affiliations:** Neuroimmunology Branch, National Institute of Neurological Diseases and Stroke, National Institutes of Health, Bethesda, MD 20892, USA; E-Mails: akahatay@ninds.nih.gov (Y.A.); JacobsonS@ninds.nih.gov (S.J.)

**Keywords:** HTLV-I, seroindeterminate, Western blot

## Abstract

Human T-lymphotropic virus type I (HTLV-I) infects an estimated 15–20 million persons worldwide. A number of diseases have been associated with the virus including adult T-cell leukemia (ATL), HTLV-associated myelopathy/tropical spastic paraparesis (HAM/TSP), HTLV-I uveitis, and HTLV-I-associated infective dermatitis. Once it was shown that there is an increased risk for developing HAM/TSP associated with blood transfusion, screening for HTLV-1 among blood banks was implemented in Japan, United States, France, and the Netherlands. This process includes detection by an enzyme immunoassay (EIA) followed by a confirmatory Western blot (WB) in which recombinant proteins specific for HTLV-I Env glycoproteins are incorporated into WB strips. HTLV-I seropositive results are defined by the presence of antibodies against either gp46 or gp62/68 (both Env protein bands) and either p19, p24, or p53 (one of the *gag* bands). HTLV-II seropositivity is confirmed by the presence of rgp46-II. However, numerous cases have been documented in which serum samples are reactive by EIA, but an incomplete banding pattern is displayed by subsequent confirmatory WB. Although the significance of these HTLV-I/II seroindeterminates is unclear, it may suggest a much higher incidence of exposure to HTLV-I/II than previously estimated.

## 1. Background

The first detection of the Human T-lymphotropic virus type I (HTLV-I) in 1980 marked the identification of the first human retrovirus [1]. While approximately 95% of HTLV-I infected individuals will remain asymptomatic for their lifetimes, roughly 5% of these patients will develop disease. Numerous diseases have been associated with the virus, including adult T-cell leukemia (ATL), HTLV-associated myelopathy/tropical spastic paraparesis (HAM/TSP), HTLV-I uveitis, and HTLV-I-associated infective dermatitis. In Japan, an estimated 1.2 million HTLV-I carriers have been identified, and more than 700 cases of adult T-cell leukemia (ATL) are diagnosed each year [2,3]. Since the initial detection and isolation of HTLV-I, HTLV research has expanded and led to the development of more sensitive and specific methods of anti-HTLV-I antibody detection. In 1984, a cell membrane immunofluorescence assay was used to detect antibodies to the HTLV-I surface protein Env. A few years later, indirect immunofluorescent assays for HTLV p19 were developed [4,5]. In 1988, radioimmunoprecipitation assays (RIPA) were used to detect these antibodies in patients who had not tested positive by other immunofluorescent methods [6]. Eventually, enzyme-linked immunosorbent assays (ELISA) became commonplace in anti-HTLV-I antibody detection, as well as the use of Western Blots (WB) to confirm infectivity [7,8]. More recently, we have developed a luciferase immunoprecipitation systems (LIPS) assay that has increased specificity and sensitivity in anti-HTLV-I antibody detection [9]. Collectively, these new methodologies have helped to shed light on the prevalence of the virus, indicating that approximately 15–20 million individuals worldwide are infected with HTLV-I. Specifically, the virus is known to be endemic in Japan, several Caribbean countries, sub-Saharan Africa, South America, and areas of Iran and Melanesia.

Among diseases associated with HTLV-I is a chronic, progressive, neurodegenerative disease termed HAM/TSP. Since the initial report of the association between HTLV-I and HAM/TSP, more associations were discovered between HTLV-I and diseases including HTLV-I uveitis, bronchopneumonitis, arthritis, polymyositis, and various other inflammatory conditions [3,10,11]. As correlations of HTLV-I with a range of medical conditions were being demonstrated, information regarding the transmission of the virus was reported as well. HTLV-I infection requires cell-to-cell contact with infected T cells. Transmission may occur via sexual contact, vertical transmission (*i.e.*, breast milk), and transfusion with infected blood [10–12]. Specifically, there is an increased risk for developing HAM/TSP associated with transfusion. In Japan, 20% of HAM/TSP patients reported a history of blood transfusion as opposed to 3% of normal donors (p < 0.001) [11].

Due to this increased risk of disease with blood transfusion, many screening methods have been employed to detect HTLV-I infected donors. Blood bank screening for the virus was implemented in Japan in 1986, the United States in 1988, France in 1991, and the Netherlands in 1993 [10,11]. These screenings were initiated once proof of increased risk for developing HAM/TSP was definitively associated with transfusion. Numerous procedures exist for anti-HTLV-I antibody detection, including ELISA, RIPA, and particle agglutination. Standard screening assays used by blood banks in the United States include initial testing by an enzyme immunoassay (EIA), followed by confirmation of seropositivity by WB [12].

## 2. Defining and Identifying Seroindeterminates

**Figure 1.**
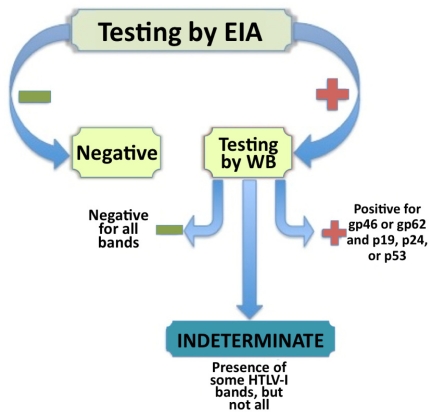
The process of identifying and classifying seroindeterminate, negative, and positive Human T-lymphotropic virus type I (HTLV-I) samples by enzyme immunoassay (EIA) testing and confirmatory Western blot (WB).

The WB used to confirm seropositivity in an EIA positive patient sample utilizes recombinant proteins specific for HTLV-I/II Env glycoproteins incorporated into the WB strips. These recombinant proteins increase the sensitivity of the blot, as well as differentiate between HTLV-I and HTLV-II. The specific sensitivity of an EIA assay is approximately 95% and the EIA specificity averages 98% while the sensitivity of the WB is 97.1% with a specificity of 97.5% [13,14]. A sample is classified as HTLV-I positive if it meets the established criteria (see Figure 1). According to the World Health Organization, an HTLV-I infected individual must have an antibody response to either gp46 or gp62/68 (both Env protein bands) and either p19, p24, or p53 (one of the Gag bands). Even more rigid criteria has been recommended by the HTLV European Research Network, suggesting bands for both Gag proteins p19 and p24 as well as the Env proteins rgp21 and rgp46-I must be present to ensure seropositivity [15].

**Figure 2.**
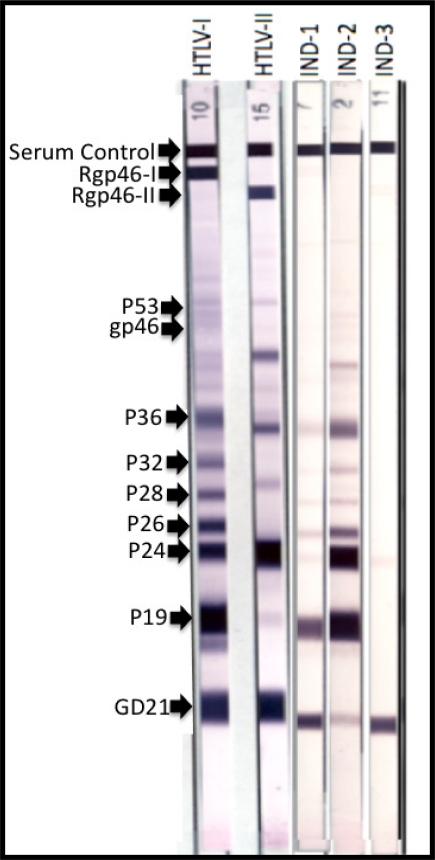
The first two Western Blot strips depict positive control HTLV-I and HTLV-II Western Blots exhibiting all core bands necessary for seropositivity inclusion, and other bands exhibited in a typical infected sample. IND-1 IND-2 and IND-3 illustrate three possible banding patterns of seroindeterminate samples.

Generally, HTLV-I infected individuals such as asymptomatic carriers, HAM/TSP patients, and ATL patients exhibit the clear seropositivity illustrated in Figure 2 on the HTLV blot 2.4 (MP Diagnostics, Solon, OH, USA). However, there have been reports around the world of HTLV-I/II seroindeterminate WB banding patterns that do not meet the strict criteria of HTLV-I seropositivity [15–21]. These are referred to as HTLV-I/II seroindeterminates (see Figure 2, blots IND-1, IND-2, and IND-3). HTLV-I/II seroindeterminate samples have been identified in otherwise healthy and normal individuals from Jamaica, Japan, Brazil, and other HTLV-I endemic areas and showed no other sign of disease or viral infection. However, of particular interest are the reports of HTLV-I/II seroindeterminate blotting patterns in patients with various neurological diseases including Multiple Sclerosis [12,22]. While the significance of HTLV-I/II seroindeterminates is still under investigation, it is clear from this finding that these samples may relate to disease (specifically neurodegenerative diseases) in at least a portion of cases. The reported prevalence of seroindeterminates has varied greatly from .023% in Taiwan, to 0.1% in Argentina, to as high as 20% of a high-risk population in Brazil [18,23,24]. Interestingly, when DNA isolated from peripheral blood mononuclear cells (PBMC) of these HTLV-I/II seroindeterminate individuals is amplified using polymerase chain reaction (PCR) assays, typically no HTLV-I or HTLV-II virus is detected (however, recent reports from Iran, Argentina, and Brazil have challenged this finding) [17]. A seroindeterminate sample is defined by a positive EIA result but an incomplete banding pattern by WB, as depicted in Figure 1 [16]. Incomplete banding patterns have manifested in various ways. A number of samples yielded all core protein bands, but lacked one or both of the recombinant bands (GD21 and rgp46-I) necessary for seropositivity (for example, IND-2 in Figure 2). Other samples seemed to exhibit a negative blotting strip except for a single protein band (as shown on IND-3 in Figure 2). Classifying a blot as seroindeterminate can vary greatly between studies and cohorts.

## 3. Possible Explanations for HTLV-I/II Seroindeterminates

As research progresses, reports of large numbers of HTLV-I/II seroindeterminates have come from Iran, Brazil, Japan, Argentina, Taiwan, the Caribbean, Central Africa, and the United States, indicating a higher prevalence than originally thought [15–21]. Importantly, the reasons for these blotting patterns remain unclear. Several possible explanations have been proposed for the occurrence of HTLV-I/II seroindeterminates that include cross-reactivities to other known retroviruses or a novel virus, antibody responses to a malaria parasite with epitope homology to HTLV-I, a defective HTLV-I or HTLV-II, and low copy numbers of prototypic HTLV-I in the affected patient yielding the indeterminate antibody response.

### 3.1. Malaria and Severe Acute Respiratory Syndrome (SARS)

It has been reported that anti-HTLV-I reactivity of sera can result from antibodies produced in response to *Plasmodium falciparum (P. falciparum). P. falciparum* is a lethal malaria parasite and is the most prevalent of malaria parasites infecting humans [14]. Studies regarding this cross-reactivity concluded that *P. falciparum* and HTLV-I must contain regions of immunogenic epitope homology. It was hypothesized that this homology may be a result of mimicry of host tissue by the two organisms [25]. This suggests that in geographic regions known to be endemic for malaria, such as the Philippines, and in which high levels of HTLV-I antibody reactivity were reported, HTLV-I/II seroindeterminates are difficult to interpret, as it is difficult to rule out the possibility of cross-reactivity between HTLV-I/II and *P. falciparum* [25]. It was later reported that this cross-reactivity might not be limited to *P. falciparum*. Examination of data from a separate cohort showed cross-reactivity with HTLV-I could be blocked by erythrocyte lysates (from areas where malaria is endemic) infected with *P. falciparum*, however HTLV-I seroindeterminate samples from the United States failed to block antibody reactivities. This led to the assertion that the molecular mimicry may extend to epitopes other than the *P. falciparum* and plasmodial antigens [26]. However, HTLV-I/II seroindeterminate banding patterns are being reported in areas where exposure to *P. falciparum* is extremely unlikely, such as the United States. Furthermore, HTLV-I/II seroindeterminate patterns are observed in normal, healthy blood donors, showing no sign of malaria or similar parasite infection [20]. While a subset of HTLV-I/II seroindeterminate samples may exhibit an antibody cross-reaction between HTLV-I and *P. falciparum*, this cannot explain the WB banding patterns found in areas where these organisms are extremely rare or nonexistent.

It should also be noted that cross-reactivity between antibodies against severe acute respiratory syndrome (SARS) coronavirus and HTLV has been observed on occasion [27]. The HTLV antibody response was exhibited when numerous serology tests were performed on samples from patients admitted to a hospital in Taiwan for SARS. However, this was immediately following the SARS outbreak in 2003, and has not been observed in other regions.

### 3.2. Cross-Reactivities to Other Retrovirus

Various other hypotheses have been presented regarding the cross-reactivity of anti-HTLV-I antibodies. In 1997, it was reported that HTLV I/II indeterminate serologies were observed as responses to two types of simian T-lymphotropic virus (STLV) [28]. An HTLV-I/II seroindeterminate sample that was also HTLV-I PCR positive was sequenced and showed a divergent form of HTLV-I [28]. Further investigations led to the suggestion that indeterminate blots may denote an antibody response to immunogenic regions of an endogenous retrovirus [29]. Also reported was the demonstration of elevated antibody levels to synthetic HTLV-I epitopes in samples previously testing negative for HTLV-I antibodies. The reactivity in this study reinforced a previous suggestion that increased levels of HTLV-I antibodies may be an autoantibody response to the endogenous retrovirus HRES-I [30,31]. The specific homologous regions between HTLV-I and these endogenous retroviruses occur in the LTR, *gag*, and *pol* regions of the virus. However, a later report demonstrated the *tax* region of prototype HTLV-I virus was amplified by nested PCR from one patient with an HTLV-I/II seroindeterminate WB that could not have been derived from the DNA sequence of an endogenous virus [12]. HTLV-I/II seroindeterminate banding patterns have also been reported in samples which were PCR positive for HTLV-I, supporting the exciting possibility that an HTLV-I/II seroindeterminate pattern may result from cross-reactivity with a novel virus such as HTLV-III or HTLV-IV [32]. These newly discovered human retroviruses were found in Cameroonese hunters showing no signs of HTLV-related diseases, and all four HTLV types show 60–70% sequence homology with each other [31].

### 3.3. Low Copy Number of Prototype HTLV-I

Due to the typically negative PCR results and lack of antibody response to some of the HTLV-I antigens but reactivity to others, the most plausible suggestions seems to be that HTLV-I/II seroindeterminate blots may result from a low copy number of prototypic HTLV-I [12]. This explanation is supported by studies showing the ability to amplify the HTLV-I *tax* region from PBMCs of some HTLV-I/II seroindeterminates, but not other regions of the virus [12,28,29]. The same study reported the successful generation of an Epstein-Barr virus transformed B-cell line from a relapsing remitting multiple sclerosis patient with a seroindeterminate WB. The PBMCs from this patient had tested negative for regions of HTLV-I by PCR, while an *in vitro* long-term generated B-cell line tested positive by primary PCR. The virus infecting the seroindeterminate B cell line was then sequenced in an attempt to identify any mutations or other factors that may be associated with an HTLV-I/II WB seroindeterminate status. The results indicated that the virus was globally >97% homologous to prototypic HTLV-I on the nucleotide level. Fine analysis of the 5′ LTR indicated that the HTLV-I strain infecting the patient was of the Cosmopolitan subtype [22]. This was the first reported verification of a PCR negative seroindeterminate sample resulting from infection of a full length HTLV-I virus [22]. Further support for the suggestion that these seroindeterminates may be the result of a low copy number of prototype HTLV-I comes from a study of patients transfused with HTLV-1 infected blood [33]. Eight seronegative individuals developed seroindeterimnate WB patterns after receiving a blood transfusion with the infected blood, further implicating the role of exposure to HTLV-I in seroindeteriminates [33].

The hypothesis that HTLV-I/II WB seroindeterminates (or subsets of these individuals) may be related to prototype HTLV-I is further supported by the recent observations that demonstrated HTLV-I PCR positive results in 12.5% of HTLV-I/II seroindeterminates from Iran [17] (Table 1). Consistent with this observation are reports from Brazil and Argentina, which also demonstrated comparable rates of PCR reactivity in HTLV-I/II seroindeterminates (9.2% and 13.8%, respectively) [17] (Table 1). The most prevalent seroindeterminate banding pattern observed in Iran was the appearance of GD21 alone, which is similar to the patterns seen in Taiwan [27,34]. The data in Table 1 also demonstrates a relatively high frequency of HTLV-I/II WB seroindeterminates from HTLV-I EIA reactive samples that are observed in multiple cohorts throughout the world. These range from 16% in the United States to 61% in Argentina. Reports of PCR amplification of prototype HTLV-I sequences from HTLV-I/II seroindeterminate individuals, especially in endemic regions such as Iran and Brazil, suggest a potentially far greater exposure to HTLV-I at least in some sub-populations of HTLV-I/II WB seroindeterminates.

## 4. Current Research and Future Directions

While the significance of these prevalent seroindeterminate HTLV-I/II blotting patterns remains uncertain, it is clear that further investigation is necessary. The sequencing of a virus with homology to prototype HTLV-I from a seroindeterminate cell line implicates exposure to the virus in at least some HTLV-I/II seroindeterminates. This finding may suggest that exposure to HTLV-I is greater than previously estimated. Of concern are reports that have demonstrated serum samples with negative HTLV-I/II results by EIA, which may also have seroindeterminate banding patterns by WB [12]. Since these sera were initially screened as HTLV-I/II EIA negative (see Figure 1), these samples (that also have an HTLV-I/II seroindeterminate WB pattern) would enter the blood supply and be available for transfusion [37]. There have also been instances of serum samples testing EIA positive and WB indeterminate at one point in time, and later testing EIA negative, while remaining seroindeterminate by WB [33]. This implies testing at one time point by EIA cannot ensure the exclusion of seroindeterminate samples. If seroindeterminate blotting patterns are a result of infection or exposure to HTLV-I (a virus associated with neurodegenerative disease, among other illnesses), and blood samples with these patterns can be negative by EIA screening and enter the blood supply, this poses a possible risk to those transfused with HTLV-I/II seroindeterminate blood although the clinical consequence of obtaining blood from an HTLV-I/II WB seroindeterminate donor is not known. Based on these observations there is a need for more stringent screening techniques for blood banks to ensure blood from seroindeterminate donors is not transfused. Increasingly sensitive and specific assays are currently being optimized to better detect anti-HTLV-I antibody responses.

One such technique is LIPS assay. The LIPS assay yields high throughput results while also generating quantitative values of anti-HTLV-I antibody responses [9]. This creates data for quantitative cohort comparison as well as allows for differentiation between HTLV-I infected disease patients and asymptomatic HTLV-I carriers and the possible generation of a risk assessment for disease development. Using antibody responses to the immunogenic epitopes of HTLV-I, Gag, Env and Tax, LIPS produces clear quantitative values for asymptomatic carriers, HAM/TSP patients, ATL patients, and may yield similar results for seroindeterminate individuals. The plasmids used in the LIPS assay are inserted with a gene including immunogenic epitope of a virus (for example, the *gag*, *env*, or *tax* region of HTLV-I). *Renilla* luciferase-fusion proteins (Ruc-antigen) are obtained from cell lysates transfected with each plasmid. A preincubated serum/antigen mixture is added to a filter plate coated with immobilized protein A/G beads. This mixture is then washed to remove any fusion proteins that did not bind to the antibody. Luciferase activity is then measured in a microplate luminometer.

The LIPS assay provides a promising high throughput method of anti-HTLV-I antibody detection. Further examination of immunogenic epitopes, such as HTLV-I Rex and HBZ, may enhance the specificity and sensitivity of the assay. Currently, a whole Gag protein is used for detection of antibody response; however, antibody responses for p19, p24, or other portion of the Gag protein may yield even more specific results. A panel of these quantitative antibody responses may be able to detect seroindeterminate samples in a fast, inexpensive, and efficient manner. LIPS may also detect seroindeterminates that tested negative by EIA, proving to be a more sensitive assay in terms of seroindeterminate detection. While the LIPS assay is under further development, other methods to improve detection and classification of seroindeterminates have also been proposed including: real-time PCR, molecular diagnostic analysis, detection of amino acid changes in the *env* region of HTLV-I, inclusion of other synthetic peptides in serological assays, and optimization of an enzyme oligonucleotide assay (EOA) [19,37–39].

With further in depth research involving these newer methodologies, the significance of these HTLV-I/II seroindeterminate banding patterns will become clearer. As it becomes apparent that the prevalence of seroindeterminates is more widespread than originally thought, it becomes increasingly important to clarify the meaning of these results. If HTLV-I/II seroindeterminates (or subsets of these subjects) represent exposure to prototype HTLV-I or HTLV-II, the frequency of infection in the general population will be greater than previously believed. It remains to be seen whether the incidence of exposure is also associated with any clinical disease outcome.

## Figures and Tables

**Figure 3.**
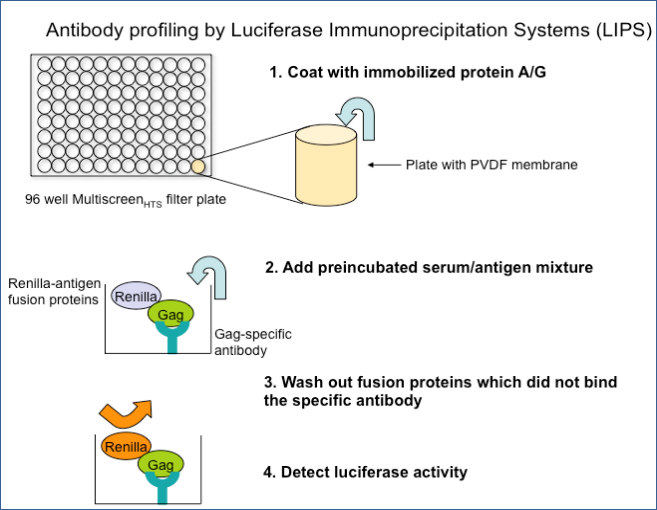
The process of antibody profiling by the luciferase immunoprecipitation systems (LIPS) assay.

**Table 1.** The table illustrates the prevalence of Human T-lymphotropic virus type I (HTLV-I) in various cohorts, as well as the percentage of HTLV-I Western blot (WB) seroindeterminates in the respective cohort and the percentage of these seroindeterimates that were HLTV-I PCR positive.

**Country [Reference]**	**Number Screened**	**HTLV-I EIA Reactive**	**HTLV-I WB + (% of Total EIA Reactive)**	**HTLV-II WB + (% of Total EIA Reactive)**	**WB Seroindeterminate (% of Total EIA Reactive)**	* HTLV-I PCR + (% of WB Serondeterminates)
Argentina [17]	86,238	0.17%	20%	8.0%	61%	13.8%
Brazil [19]	351,639	1.22%	24%	Not Shown	52.2%	9.2%
Iran [17]	79,687	6.4%	50.7 %	3.1%	25.4%	12.5%
Taiwan [35]	1,122,879	0.1%	41%	1.0%	22%	Not Shown
US [36]	267,650	0.89%	3%	Not Shown	16%	0.00%

* PCR results were obtained from a subset of WB Indeterminate samples for each cohort, as all samples were not available for further analysis.
